# Progress on Separation and Hydrothermal Carbonization of Rice Husk Toward Environmental Applications

**DOI:** 10.1002/gch2.202300112

**Published:** 2023-07-19

**Authors:** Hiroya Abe, Yuta Nakayasu, Kazutoshi Haga, Masaru Watanabe

**Affiliations:** ^1^ Frontier Research Institute for Interdisciplinary Sciences (FRIS) Tohoku University 6‐3 Aoba, Aramaki, Aoba‐ku Sendai 980–8578 Japan; ^2^ Graduate School of Engineering Tohoku University 6‐6‐11 Aoba, Aramaki, Aoba‐ku Sendai 980‐8579 Japan; ^3^ Graduate School of International Resource Sciences Akita University 1‐1, Tegata‐Gakuenmachi Akita 010‐8502 Japan

**Keywords:** biomass, hydrothermal carbonization, rice husk

## Abstract

Owing to the increasing global demand for carbon resources, pressure on finite materials, including petroleum and inorganic resources, is expected to increase in the future. Efficient utilization of waste resources has become crucial for sustainable resource acquisition for creating the next generation of industries. Rice husks, which are abundant worldwide as agricultural waste, are a rich carbon source with a high silica content and have the potential to be an effective raw material for energy‐related and environmental purification materials such as battery, catalyst, and adsorbent. Converting these into valuable resources often requires separation and carbonization; however, these processes incur significant energy losses, which may offset the benefits of using biomass resources in the process steps. This review summarizes and discusses the high value of RHs, which are abundant as agricultural waste. Technologies for separating and converting RHs into valuable resources by hydrothermal carbonization are summarized based on the energy efficiency of the process.

## Graphical abstract

This review summarizes and discuss the high value of rice husks based on the energy efficiency of the process, technologies for separating and converting RHs into valuable resources by hydrothermal carbonization. In addition, the application as energy‐related and environmental purification materials such as battery, catalyst, and adsorbent via hydrothermal carbonization are summarized.

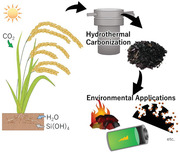

## 1 Introduction

The growing global demand for carbon resources is increasing pressure on finite petroleum and inorganic material resources. Similarly, greenhouse gas carbon dioxide emissions are increasing every year and need to start decreasing before 2025 to return to pre‐industrial emission levels.^[^ ^1^ ^]^ In addition, international instability, including global infectious diseases, has renewed the focus on using local resources in manufacturing. To address this environmental problem, using alternative energy sources, such as biomass, to replace fossil fuels is essential for a sustainable society.

Biomass is a concept that expresses the quantity (mass) of biological resources (bio) and refers to organic resources (excluding fossil resources such as oil and coal) derived from plants and animals that can be recycled into energy and materials, such as agricultural and forestry products, rice straw and husks, and food, animal, and wood waste.^[^ ^2^ ^]^ However, a balance between supply and demand is established in the production and consumption of biomass, and excessive consumption is expected to damage biodiversity and the environment severely. For example, wood biomass is being actively researched for use in various energy and environmental materials because of its porous nature,^[^ ^3^, ^4^, ^5^, ^6^, ^7^, ^8^, ^9^ ^]^ but potential deforestation must simultaneously be considered. The effective use of waste biomass resources is therefore needed.^[^ ^10^, ^11^ ^]^ For example, citrus pulp can be converted to bioethanol, hesperidin, and nanocellulose as value‐added products,^[^ ^12^ ^]^ and activated carbon (AC) derived from banana peels performed well in the development of a supercapacitor owing to its hierarchical porous carbon structure.^[^ ^13^ ^]^ The efficient use of waste resources is crucial for sustainable resource acquisition for creating the next generation of industries.

Rice husk (or rice hull) (RH) is an abundant agricultural waste produced from rice plants (*Oryza sativa*) and contains lignocellulose and silica. In particular, the high silica content is a distinguishing characteristic compared to other biomass types, resulting in a substantial difference from the typical utilization of lignocellulosic biomass. Raw RH is used in environmental applications such as adsorbents for adverse heavy metals and organic pollutants^[^ ^14^ ^]^ and biofuels for generating energy.^[^ ^15^ ^]^ However, the effective use of RH is challenging owing to its unique chemical and physical properties, such as its hard surface, high silica content, and low feed value. Moreover, RH is strongly resistant to decomposition by microorganisms in the soil. Thus, there is an urgent need to convert raw RH into value‐added products.

Currently, there are three approaches to the use of RH, including 1) separation of silica and carbon for independent utilization, 2) focusing only on the carbon component without removing the silica, and 3) using RH to take advantage of its silica content. In many cases, the conversion process consumes a large amount of energy; therefore, saving energy during the conversion process reduces environmental pressure.

The Berl and Schuhmacher groups investigated the hydrothermal carbonization (HTC) process, which was developed by Bergius in 1913,^[^ ^16^ ^]^ for assessing the biomass source,^[^ ^17^ ^]^ the formation process,^[^ ^18^ ^]^ and the identification of the final hydrochar composition.^[^ ^19^ ^]^ The HTC process provides several benefits, including 1) the HTC temperature is usually much lower than that of pyrolysis, gasification, and flash carbonization; 2) there is no necessity for pre‐drying as water is used as the solvent; and 3) some gases, such as CO_2_, nitrogen oxides, and sulfur oxides, are dissolved in water, forming the corresponding acids and salts, making further treatment for air pollution possibly unnecessary.^[^ ^20^ ^]^

**Figure 1:**
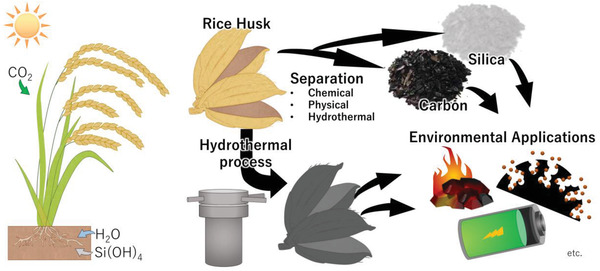
Value‐added rice husk (RH) via separation and hydrothermal carbonization (HTC) processes.

This review summarizes recently developed HTC processes for the separation and conversion of RH (**Figure** **1**). We first introduce the basic properties of RH and the fundamental components of HTC. Next, the separation process of RH into carbon and silica using the conventional and HTC processes is reviewed and discussed. Finally, we review and discuss recent environmental applications of RH using conventional and HTC processes.

## 2 Characteristics of Rice Husk

Rice is produced worldwide and is divided into four major types: japonica, indica, aromatic, and glutinous. The characteristics of these types of rice are different in terms of size, taste, color, and composition. According to Food and Agriculture Organization of the United Nations (https://www.fao.org/faostat/en/#data/QCL), in 2020, 756.7 million tons of rice grains were produced from 115 countries. China and India contribute more than 50% of the global rice production, producing ≈214 and 178 million tons of rice per year, respectively. In addition, Asia accounts for 89% of global rice production. The RH comprises ≈20% of the total grain weight; therefore, the global RH production is ap≈150 milion tons. Although there are many varieties of rice, their composition is roughly similar.

**Figure 2:**
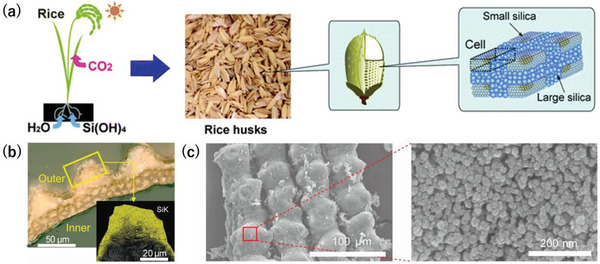
a) Images of silica inside the RH. The image was adapted and modified from ref. [27] with permission from the Royal Society of Chemistry, copyright 2010. b) Optical microscope image of an RH shell and silicon mapping (inset). c) Scanning electron microscopy images of silica derived from the RH. Images (b and c) were adapted and modified from ref. [28] with permission from the National Academy of Science, Copyright 2013.

The RH consists of silica (15%–20%), lignin (20%–30%), holocellulose (55%–65%), and others (2%–5%); thus, the RH is a mixture of ≈80% organic and 20% inorganic components (**Figure** **2**a). The main component of inorganic components consists of silica, and other minerals such as potassium and calcium are contained, whose oxides are remained as ash.^[^ ^21^ ^]^ Several plants, including rice, accumulate silicon as silica nanoparticles. Silica improves the strength of rice against wind and rain and protects against destructive insects;^[^ ^22^, ^23^, ^24^ ^]^ therefore, irrigation water has long been fortified with silicate to improve the properties of rice plants. The silicon component is transported via a silicon transporter (*Lsi1*)^[^ ^25^ ^]^ and delivered into the cell.^[^ ^26^ ^]^ Figure 2b indicates the abundant silica in the RH outer cell wall. Tabata et al. reported that RH nanostructures calcined in nitrogen gas at 800 °C.^[^ ^27^ ^]^ Closely packed silica nanoparticles were observed on the surface of the cells (Figure 2c). The specific surface areas of RH before and after the leaching of silica with hydrofluoric acid (HF) solution were 40 and 612 m^2^ g^−1^, respectively. In addition, the pore size of the leached RH was 50–100 nm, indicating that the silica nanoparticles in RH have potential as a template for nanoporous carbons. Additionally, RH silica is a potential energy material. Jun et al. developed lithium battery anodes converted from RH.^[^ ^28^ ^]^ Because RH silica comprises nanoparticles under 100 nm, the use of MgO in the reduction of 3D porous silicon demonstrated superior performance.

Upon review of its properties, RH was found to possess considerable potential as a nanoporous carbon and inorganic nanomaterial; however, to separate arbitrary materials, the removal processes of either carbon or silica, such as HF etching during the leaching process, consumes a high amount of energy and places substantial pressure on the environment. Therefore, the conversion process should be reframed by assessing environmental impacts and identifying mitigation measures for energy consumption.

## 3 Hydrothermal Carbonization Process

Biomass, such as agricultural residues, livestock manure, and food waste, contains high amounts of water and has been converted into resources and energy through composting. The upgrading process technologies have rarely been linked together as a single system because of the different characteristics of dry and wet biomass. Each technology requires considerable energy to pre‐dry biomass, which wastes gas. Biomass containing over 70% water can be used as the precursor for the hydrothermal carbonization (HTC) process and saves energy by skipping the pre‐drying process in the conversion of wet biomass into high‐quality char (hydrochar).

**Figure 3:**
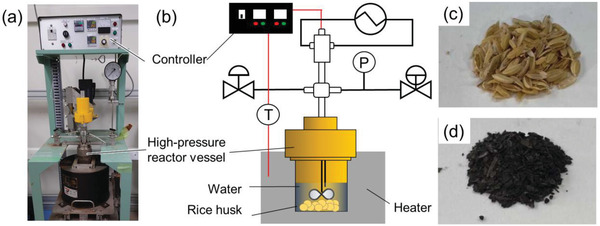
a) Photograph of a hydrothermal vessel. b) Diagram of the hydrothermal vessel. c and d) Photographs of RH before and after hydrothermal treatment (HTC), respectively.

Batch type equipment is often used for HTC at the laboratory level. Reaction vessels made of strainless materials are often used because of their thermal conductivity and pressure resistance (**Figure** **3**a,b). Biomass raw material is placed directly into the reactor without going through the drying process, and water is heated externally by heaters such as an electric furnace.^[^ ^20^ ^]^ The pressure inside the reactor increases mainly because of the water vapor pressure, resulting in a high‐temperature and high‐pressure environment exceeding the standard boiling point of water (100 °C). The color of HTC biomass often changes to brown or black through browning reactions, such as caramelization, to form multi‐ring hydrocarbons, including heteroatoms such as O and N (Figure 3c,d). In several cases, hydrothermal treatment of biomass with high moisture content is possible using only the water content in the biomass. Lignocellulose, the main constituent of plant‐derived biomass, has strong crystallinity owing to hydrogen bondsand is therefore commonly converted into manageable biomass by HTC decomposition and leaching. In addition to the hydrolysis reactions, pyrolysis and polycondensation reactions also occur during the hydrothermal process at low temperatures. Oxygen‐containing functional groups are first decomposed at low temperatures into gases via dehydration and polymerization, leading to an increase in the calorific value of the remaining solid (hydrochar). The carbonized substances from HTC include charred materials resulting from the polycondensation of water‐soluble decomposition products. The resulting biochar is also less hydrophilic and often forms slurry‐like solids.

**Figure 4:**
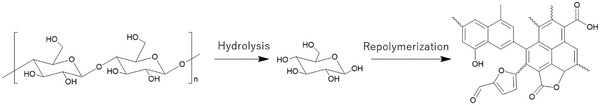
Hydrolysis and carbonization of cellulose with HTC.

Although the hydrothermal process is complicated, its mechanisms have been well‐studied.^[^ ^20^, ^29^, ^30^ ^]^ Fragmentation and dissolution of the amorphous phase (lignin and hemicellulose) of willow was achieved easily at temperatures as low as 200 °C.^[^ ^31^ ^]^ The HTC of cellulose results from a temperature rise to 220 °C as shown in **Figure** **4** and the samples consist mainly of aggregates of microspheres with a diameter in the 2–10 µm range.^[^ ^32^ ^]^ Meanwhile, the products from hydrothermal treatment at 210 °C exhibited an irregular morphology similar to that of pristine cellulose.^[^ ^32^ ^]^ However, the HTC process requires a long residence time, which may lead to polymerization of soluble intermediates in the liquid phase and peculiar microstructural characteristics of the hydrochar.^[^ ^33^, ^34^ ^]^ Therefore, how to rapidly obtain semi‐cabonized materials with high calorific value and high carbon content under simple and mild conditions is an urgent issue.

Recently, catalytic hydrothermal carbonization with the addition of acids is known for upgrading the properties of hydrochars. Acid‐catalyzed HTC promotes rapid degradation of lignocellulosic components, especially cellulose and hemicellulose.^[^ ^35^ ^]^ The syntesized hydrochars have shown higher heating value (HHV), lower ash content, improved combustion characteristics, and higher energy yield compared to hydrochars produced without organic acids.^[^ ^35^ ^]^ For example, Ru et al. found that citric acid increased HHV from 20.3 to 27.7 MJ kg^−1^ and energy yield from 19.2% to 82.9% by catalytic hydrothermal carbonization of pomelo peel.^[^ ^36^ ^]^ Xu et al. investigated the catalytic role of NaCl for HTC and found that the NaCl promoted the hydrolysis, dehydration and decarboxylation performance.^[^ ^37^ ^]^

In addition, surfactants with amphiphilic molecular structures have been used to form water/oil dispersion systems, which can have diverse biomass conversion pathways by lowering the surface tension between water and oil during the HTC process.^[^ ^38^, ^39^ ^]^ The addition of a surfactant to the acid‐catalyzed HTC system probably react with the degraded components in the surfactant domain to form a hydrotchar with improved carbonization strength and surface properties.^[^ ^40^ ^]^ In fact, addition of Span 80 as a surfactant to citric acid‐catalyzed HTC significantly increased the mass yield of hydrothermal carbide from 39.04% to 63.45% and HHV from 24.122 to 31.302 MJ kg^−1^, respectively.^[^ ^41^ ^]^

Application to lignocellulosic biomass is less effective due to low yields and low porosity resulting from excessive degradation of the organic substrate. In this regard, HTC‐treated biomass is characterized by a more “coal‐like” chemical structure and thus it is a more suitable precursor for the production of highly porous AC.^[^ ^42^ ^]^ Generally, the porous structures are effective for the electrode materials such as supercapacitors, electrode catalysts and supporing materials. The process of fabricating carbon‐based electrode materials is viewed as 1) precursor preparation (including metals impregnation treatment, etc.); 2) main heat treatment (vapor phase carbonization, etc.); 3) activation (chemical or gas activation treatment); 4) acid or alkali purification.

## 4 Separation Technology for Carbon and Silica Components

### 4.1 Effectiveness of Separation Technology

RHs contain ≈20% inorganic materials in addition to carbon,^[^ ^43^ ^]^ and the most of component is silica.^[^ ^21^ ^]^ Hence, when RHs are directly utilized as fuel, silica remains as ash, which is ≈20% of the supply weight. In addition, when used as a raw material for carbon or silica, RHs are challenging to use directly due to many impurities. Therefore, an effective technology for separating the carbon component and silica from RHs is needed to increase the value of biomass resources.

The possibility of chemical and physical separation methods for carbon and silica from organic resources containing silica, such as RH and its ash, are discussed in this section. **Table** **1** summarizes the separation methods and the role of technology for RHs in this study. Methods for recovering carbon and silica from organic resources can be broadly classified into chemical separation by wet treatment, physical separation based on differences in the physical properties of the particles, and thermal treatment. Examples of research on the separation of carbon and silica by wet processing using acids and alkalis are introduced as chemical separation methods. No physical separation studies have been performed on RHs. This section includes a review of research examples on the physical separation of silica‐containing organic resources, such as coal and coal ash, and prospects for the physical separation of RHs are discussed. In addition, this paper describes the dry heating and hydrothermal heat treatment methods focused on in this study, and their roles in separation and expectations for the future are explained.

**Table 1:** Methods of RH separation

Separation		Mechanism	Ref.
Wet chemical treatment	Acid treatment (e.g., HCl)	Hydrolysis of lignocellulose, removal impurities (metal, salts)	[44, 45, 46]
	Alkali treatment (e.g., NaOH)	Dissolution of ash precursors (silica)	[44, 45, 46]
	Ionic liquid	Extraction of lignocellulose and silica by dissolution of lignocellulose into ionic liquid	[47, 48]
Thermal treatment	Dry heat treatment	Decomposition of organic substance	[49]
	Hydrothermal leaching	Dissolution of ash precursors, carbonizing organic substance	[50]

### 4.2 Wet Chemical Separation

Wet treatment studies have assessed the use of acid or alkali to remove impurities from RHs or selectively separate and recover silica. Acid treatments are mainly used to remove metallic impurities.^[^ ^43^ ^]^ Impurities such as silica, aluminum, iron, and magnesium, are present in RHs in trace amounts. When RHs and straw are used as a carbon source for biochar and other products, silica, salts, and metals are considered impurities and are dissolved with hydrochloric acid, sulfuric acid, and organic acid.^[^ ^37^, ^38^, ^39^ ^]^ However, many studies have utilized calcined RH ash to volatilize the carbon content when RHs are used as a source of silica. The incinerated RH ash is high in silica and can be used as a raw material in products such as cement and bio‐silica (green silica). In this case, removing the coexisting impurities and increasing the silica content will yield higher‐quality materials thus research is being conducted on the removal of impurities by acid leaching using hydrochloric acid, sulfuric acid, and carboxylic acids, including citric acid, acetic acid, oxalic acid, and gluconic acid.^[^ ^40^, ^41^, ^42^, ^43^, ^44^, ^45^ ^]^

Various studies have assessed alkali treatment as an effective process for the leaching and removal of inorganic components. Alkali treatment is also a common chemical separation method for silica and carbon. Carbon and silica can be activated and separated simultaneously when applied to organic‐inorganic mixed materials such as RHs.^[^ ^51^ ^]^ The selective separation of carbon and silica is necessary to improve the biomass value. Silica, the main ash component, dissolves under strongly alkaline conditions, such as potassium hydroxide and sodium hydroxide, thereby improving the biomass quality.^[^ ^52^ ^]^ Treatment with a sodium hydroxide solution with a pH of over 10 solubilizes amorphous silica in RHs.^[^ ^53^ ^]^ Several studies have used this sodium hydroxide solution to remove toxic elements, such as cadmium and arsenic, in RH ash.^[^ ^54^ ^]^ When separating silica from a solution by alkaline leaching, silica precipitate can be obtained by blowing and neutralizing the solution with carbon dioxide.^[^ ^55^ ^]^ Alkaline leaching has also been applied in several cases for the selective separation of lignin from biomass because lignin dissolves in alkaline conditions compared with cellulose and hemicellulose, which do not.^[^ ^56^ ^]^ In addition, silica removal by adding potassium hydroxide (KOH) as an alkali activates RH‐AC, improving its functionality as a biomass material.^[^ ^57^ ^]^

On the other hand, it is also possible to separate silica by dissolving organic materials instead of dissolving silica. Among them, the process using ionic liquids (ILs) is well known. By pyrolyzing the RH residue after extraction with ILs, amorphous silica nanoparticles with high purity, high surface area, and small particle size distribution have been successfully synthesized.^[^ ^47^ ^]^ By combinating dicationic ionic liquids with sonication has resulted in 26% lignin extraction. Furthermore, the ILs did not lose its performance even after three repeated uses and could be recycled 90% of the time. Extraction using ILs is highly advantageous for commercialization because no heating energy is required, it does not take long time, and ILs can be easily recycled for up to several cycles.^[^ ^48^ ^]^

### 4.3 Thermal Processes

#### 4.3.1 Dry Heat Treatment

Silica can be extracted from RH by heating it under ambient conditions. The carbon content in RH can be decomposed into CO_2_ and H_2_O at high temperatures. The nanostructure of the silica is retained even after heating to 800 °C, indicating that the heat is suitable for removing carbon and obtaining good‐quality silica.^[^ ^49^ ^]^ However, a large amount of energy and carbon content are lost via the heating process, which is not eco‐friendly or economical; thus, an alternative method that meets these requirements is needed in RH.

#### 4.3.2 Hydrothermal Leaching Process with HTC

Among inorganic compounds, metal oxides can be leached by hydrothermal treatment.^[^ ^58^, ^59^ ^]^ Silicon in RHs exists in the amorphous SiO_2_ state. The dissolution of amorphous silica in water can be represented by the following reaction, with H_4_SiO_4_ as the dominant aqueous silica species:

(1) $$\begin{array}{c} {\mathrm{SiO}}_{2}\left(s\right)+2H_{2}O\left(1\right)=H_{4}{\mathrm{SiO}}_{4} \end{array}$$

This amorphous silica dissolves well in subcritical water above 100 °C.^[^ ^50^ ^]^ In the RH HTC process, the dissolution reaction of silica is accelerated, which produces high‐quality carbon and has the advantage of simultaneous separation. There are few reports on separation using hydrothermal processes. Nazia et al. observed that the SiO_2_ content of RHs decreased from 27.44% to 13.46% after the HTC reaction. However, the ratio decreased for other types of ash.^[^ ^60^ ^]^ As described in Section 3, the hydrothermal process provides several benefits, including low environmental impacts, low costs, and high‐quality materials; therefore, the separation process using HTC will become a prevailing separation process.^[^ ^61^ ^]^

### 4.4 Potential of Physical Separation

The physical process is environmentally friendly because it does not use costly and environmentally hazardous reagents such as acids and alkalis. The silica contained in RHs is challenging to physically separate because it is finely and uniformly dispersed. No studies have been conducted on the physical removal process of RH silica.

This section provides examples of research on the physical separation of silica from solid carbon‐containing resources, such as coal, and the associated incinerated bottom ash and fly ash. These examples provide a possible eco‐friendly process for separating carbon and silica. The substantial difference between the physical properties of carbon and silica is attributed to their specific gravity and hydrophobicity. The specific gravity of high‐grade coal is ≈1.4 to 1.6, whereas silica is ≈2.5. Therefore, it is possible to separate each material using specific gravity differences.^[^ ^62^ ^]^ Typical separation methods that utilize specific gravity differences include gravity and centrifugal separation. In addition, the surface of carbon is hydrophobic and silica is hydrophilic; thus, separation of materials is also possible because of the difference in wettability. Hence, flotation is used as a typical method for separation based on the differences in wettability.

## 5 Application of RH‐Derived Materials using HTC

The products obtained by carbonizing RH in a nitrogen atmosphere have been used in various materials and applications, such as fuels, absorbents, and energy‐related materials. The HTC process converts biomass at relatively low temperatures into a carbon‐rich energy‐dense solid, which can be used as a solid fuel with an energy density equivalent to coal.^[^ ^63^ ^]^

This chapter describes the RH‐derived materials synthesized using HTC in recent years. In addition, the materials and applications synthesized by existing carbonization methods in recent years, as well as those synthesized by applying HTC to other biomass types, are also presented, and the potential applications of RH‐derived materials using HTC methods are summarized.

### 5.1 Fuel

Because of the low environmental impact of the upstream process and the use for fuel production through multiple conversion technologies, RH is the biomass feedstock of choice to replace fossil fuels for immediate and sustainable reduction of greenhouse gas (GHG) emissions.^[^ ^64^, ^65^, ^66^ ^]^ Three treatment pathways can convert RHs into energy: HTC, catalytic pyrolysis, and anaerobic digestion. These require different treatment pathways that are economically and environmentally advantageous.

Zhu et al. investigated the effect of temperature on carbon quality during the pyrolysis of cellulosic biomass.^[^ ^67^ ^]^ *S. psammophila* was used as the carbon resource. The cellulosic biomass was pyrolyzed at temperatures ranging from 300 to 700 °C for 4 h. The O/C and H/C atomic ratios of the obtained char decreased with increasing temperature, indicating that temperature largely contributed to the biochar quality. Furthermore, the dehydration reaction during thermal treatment reduced both atomic ratios.

From the viewpoint of both GHG emissions and cost‐effectiveness, the HTC process is superior among the upgrading processes for producing fuel.^[^ ^68^ ^]^ Numerous practical cases of HTC using RHs as feedstock have been demonstrated. A previous study addressed waste RH utilization for good RB mass yield, 57.9%) and energy yield, 80.8% through the HTC approach.^[^ ^60^ ^]^ A microwave assists the HTC. Nizamuddin et al. achieved the hydrochar yield of 62.8% by combining HTC with the microwave at 1000 W of electric power (220 °C) for 5 min.^[^ ^69^ ^]^ Zulkornain et al. optimized microwave‐assisted HTC and achieved the highest solid yield (81.7%) at 150 °C for 10 min.^[^ ^70^ ^]^

**Figure 5:**
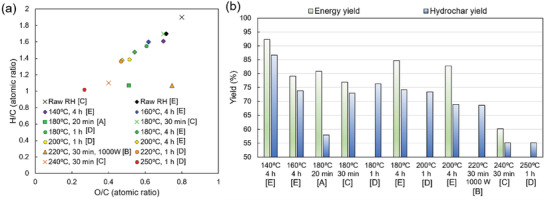
a) Van‐Krevelen diagram from previous studies. b) Energy yields and hydrochar yields under different conditions. The data values of A, B, C, D, and E are reproduced from ref.[60, 64, 65, 66, 69] respectively.

The O/C and H/C atomic ratios of RH‐derived hydrochars were plotted in recent years on the Van Krevelen diagram, as shown in **Figure** **5**a. The van Krevelen diagram is useful for clarifying solid‐state fuel properties. The graph plots better solid‐state fuels close to the origin.^[^ ^71^ ^]^ In general, the raw RH was located in the upper right corner of the Van Krevelen diagram, and the hydrochars moved toward the origin as the HTC temperature increased. At the lowest feedwater pH, the hydrochars were even closer to the origin, indicating that the fuel quality of these hydrochars was superior.

For HTC above 240 °C, the O/C and H/C atomic ratios were notably closer to the origin, whereas the hydrochar yield decreased, as shown in Figure 5b. Therefore, a processing temperature of ≈180–200 °C is suitable for this process, which satisfies both energy yield and solid fuel qualities. In contrast, the H/C ratio was lower, and the O/C ratio was higher when treated with microwaves.^[^ ^69^ ^]^ Microwaves assist in faster RH dehydration than conventional HTC, whereas the decarboxylation reaction may not be advanced.^[^ ^72^ ^]^

### 5.2 Adsorbent

Adverse heavy metals and organic pollutants can be absorbed by RH and its additives. The presence of harmful heavy metals and organic compounds in drinking water, river water, and seawater within the range of living organisms can have substantial negative impacts on humans, animals, and plants. For example, mercury released from a factory into a river caused severe damage to public health in Minamata, Japan. In addition, the kidneys, skeletal system, and respiratory system are affected by Cd^2+^, which is a human carcinogen. Because of its silica nanostructure, RH is expected to be an adsorbent. Ajmal et al. reported the synthesis of phosphate‐treated RHs (PRH) synthesized from RH,^[^ ^73^ ^]^ which performed well as an absorbent of heavy metals, comprising Ni^2+^, Cd^2+^, and Pb^2+^. Deshmukh et al., reported the investigation of removal efficiency of fluoride by RH‐derived ACs as adsorbents.^[^ ^74^ ^]^ RHs also function as adsorbents for harmful organic gases, e.g., aldehydes^[^ ^75^ ^]^ and valuable substances such as petroleum and kerosene.^[^ ^76^ ^]^ Calcining RH under ambient or inert atmospheres produces RH ash (RHA), which can recover heavy metals, such as lead, mercury, and zinc from water. Swarnalakshmi et al. reported the removal of organic compounds such as acid orange 7, amoxicillin, metformin, and carbamazepines by RHA.^[^ ^77^ ^]^ The efficient removal of RHA from water and the adsorption properties of TiO_2_ owing to its catalytic function were observed.^[^ ^78^ ^]^

ACs with a high specific surface area can be produced using HTC. This large specific surface area can be used to create ACs, which can adsorb pesticides and microorganisms in soil and promote their biological decomposition. The present study demonstrates that it is possible to produce RH hydrochars as effective adsorbents for removing harmful organics and metal ions from aqueous solutions via sorption.^[^ ^79^, ^80^, ^81^, ^82^, ^83^ ^]^ Li et al. demonstrated the synthesis of hydrochars derived from RH through metal‐chloride‐assisted HTC to adsorb metal ions and organic components.^[^ ^82^ ^]^ Setianingsih et al. synthesized MFe_2_O_4_/CNS (M = Zn, Ni, Mn) composites derived from RH using the hydrothermal microwave method for the remediation of paddy fields.^[^ ^84^ ^]^ The composites successfully removed the pesticide solution (0.25% buthylphenylmethyl carbamate). Hossain et al. examined the preparation of RH‐based porous materials with KOH activation and Ag nanoparticle incorporation for wastewater treatment.^[^ ^85^ ^]^ The hydrochar surface area was improved by KOH activation, and the Ag nanoparticles improved the zeta potential of the obtained hydrochars. More recently, RH hydrochars have been used as carbon dioxide adsorbents, methane adsorbents as energy sources, and hydrogen adsorbents.^[^ ^86^, ^87^ ^]^

### 5.3 Electrodes Materials

The development of highly efficient energy storage devices is urgently required to use green energy technologies, such as wind turbines and photovoltaic power generation. The recent progress in the application of RH to energy devices with and without the HTC process is summarized in this section.

#### 5.3.1 Li‐Ion Battery

Lithium‐ion batteries (LIBs) are commercialized storage batteries used in various fields, including electronic and electrical devices such as cellular phones and laptop computers, consumer energy storage devices, and electric vehicles. Further capacity increases, and technological innovations are underway. In particular, developing LIB electrodes composed of abundant, environmentally friendly, and renewable materials will significantly contribute to achieving sustainable energy. However, the studies on the stable supply and environmental compatibility of LIB anode materials are limited. Graphite is safe and durable and is commonly used as an anode material for LIBs, which has a theoretical capacity of 372 mAh g^−1^ due to the formation of LiC_6_, owing to its ability to transfer Li‐ions at low voltages. Recently, highly crystalline carbon for negative electrodes has been synthesized from biomass by using iron‐catalytic graphitization. Normally, when biomass is heated in an inert gas atmosphere, it only becomes hard carbon, while it is known to have a graphitic carbon structure with an iron catalyst.^[^ ^5^, ^88^, ^89^, ^90^ ^]^ In addition, graphitic carbon can be produced by annealing hydrochar with HTC as a pre‐treatment.^[^ ^91^, ^92^ ^]^ In recent years, silicon‐based anodes with a high capacity of ≈4200 mA h g^−1^ and a relatively low discharge potential plateau of ≈0.4 V versus Li^+^/Li upon full lithiation with the formation of Li_22_Si_5_ have also attracted attention.^[^ ^93^, ^94^ ^]^ The good environmental compatibility, low toxicity, and relatively stable chemical properties render silicon as a promising candidate for next‐generation LIB anodes.

**Figure 6:**
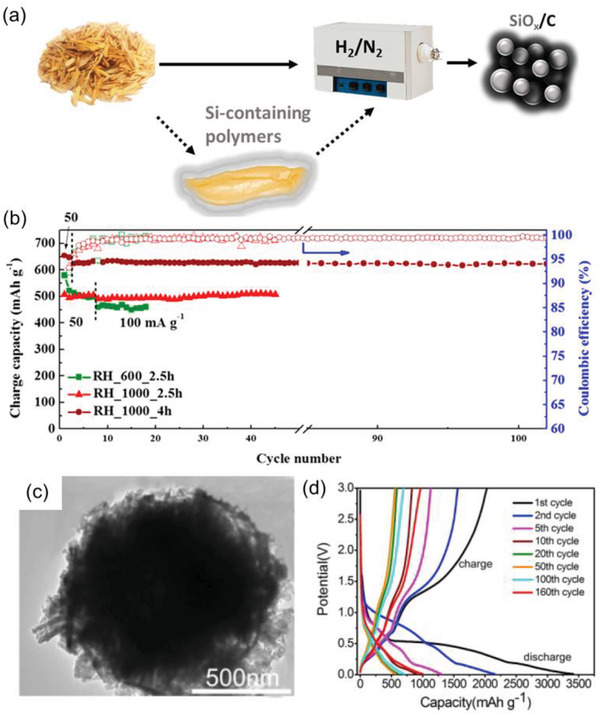
a and b) Graphical scheme of the synthesis SiOx/C from RH and the electrochemical performance of a Li‐ion battery (LIB), respectively. The images were adapted from ref. [106] with permission from the American Chemical Society, Copyright 2019. c and d) Scanning electron microscope (SEM) images of a carbon‐coated ZnO particle and electrochemical performance of the LIB, respectively. The images were adapted from ref. [115] with permission from Elsevier, Copyright 2020.

In 2003, Jung et al.^[^ ^28^ ^]^ and Liu et al.^[^ ^95^ ^]^ investigated valuable RH‐derived energy materials. Carbon and silicon, integral components of RHs, play essential roles in electrochemical energy storage devices. A high‐capacity LIB anode material was obtained to synthesize RH silicon nanoparticles using magnesio and alumino thermal processes. The theoretical capacity of silicon is ≈4000 mAh g^−1^, which is approximately ten times higher than that of conventional graphite anodes (≈370 mAh g^−1^). Liu et al. reported that the silicon anodes achieved 2790 mA h g^−1^ and long cycle life (86% capacity retention over 300 cycles). Smaller specific capacity was observed in SiOx than in pure Si, but it has a more minor volume expansion and a more stable electrode‐electrolyte‐interface phase (SEI).^[^ ^96^ ^]^ As a result, SiOx can maintain better cycle stability than pure silicon when used as an LIB anode.^[^ ^97^, ^98^, ^99^, ^100^, ^101^, ^102^, ^103^ ^]^ J. Chen et al. fabricated Si/SiO_X_@C nano‐/microstructured spheres from RH and reported that although volume expansion of Si occurs, a thin SEI film was formed that is well stable even after multicycle charge‐discharge measurements.^[^ ^104^, ^105^ ^]^ This is due to the fact that SiO_x_ built up by thermal decomposition with ionic liquids and organic materials buffered the Si volume expansion, pitch‐derived soft carbon formed stable Si—O—C, Si—C, and Si—N bonds at the interface, which further reduced the expansion of Si nanoparticles during cycling, and ionic liquid assisted in the formation of the graphitized structure, which is believed to be due to the excellent layered graphitized structure of the outer shell, which significantly improved the electrical conductivity of the anode material. Wu and Kumagai groups took advantage of the silica and organic components contained in RH and used a one‐step pyrolysis process to convert RH to an SiOx/C composite, which is an LIB electrode material.^[^ ^106^, ^107^ ^]^ The RH‐derived SiOx/C composites were synthesized through pyrolysis at RH 1,000 °C for 4 h, and the SiOx/C anode achieved 654 mAh g^−1^ and retained 88% capacity (99.8% CE) after 1000 cycles (**Figure** **6**a,b). The group also performed a full cell of Li‐ion sulfur battery incorporating the SiOx/C anode and sulfur–polyacrylonitrile cathode. The full cell showed 1566 mAh g^−1^‐S (at 160 mA g^−1^) at a first cycle reversible capacity and retained 706 mAh g^−1^‐S at 1600 mA g^−1^ after 1000 cycles. Guo et al. produced a long‐cycle life LIB anode using a silicon oxide‐embedded nitrogen‐doped carbon superstructure with ultrafine metal (Fe, Co, and Ni) nanoparticles.^[^ ^108^ ^]^ The Ni/SiOx/nitrogen‐doped carbon was synthesized from metal sources, RHs, and polyacrylonitrile, and the resultant LIB anode demonstrated 757.5 mA h g^−1^ at the 200th cycle. Recently, RH‐derived anode binder was developed by Kumagai group.^[^ ^109^ ^]^ RH‐derived‐anodes have been applied to potassium^[^ ^110^, ^111^ ^]^ and sodium‐ion batteries^[^ ^112^ ^]^ because of the abundance of Na and K resources.^[^ ^105^ ^]^

Biomass‐derived anodes were synthesized using HTC and RHs could be upgraded to carbon nanomaterials possessing a high surface area, which increased the performance of the LIBs while saving energy for conversion. M. Titirici et al., formed RHs‐derived carbon for lithium‐ion battery anode by using HTC process as a pretreatment.^[^ ^113^ ^]^ Yu et al. demonstrated the synthesis of zinc‐cobalt bimetallic oxide anchored on the surface of RH carbon as an anode for a high‐performance LIB through a hydrothermal process.^[^ ^114^ ^]^ The obtained composites zinc‐cobalt bimetallic oxide and RH carbon composites showed a high charge capacity of 972 mAh g^−1^ over 150 cycles at 100 mA g^−1^. The RH carbon improved the electronic conductivity of zinc‐cobalt bimetallic oxide nanoparticles and reduced the deterioration in performance because of volume expansion during charge and discharge. Li et al. demonstrated the synthesis of RH hollow carbon‐coated flower zinc oxide as an anode in high‐performance LIBs.^[^ ^115^ ^]^ A composite precursor of ZnO and RH‐cellulose was prepared using HTC at 200 °C for 4 h (Figure 6c,d). The zinc oxide and carbon composite demonstrated a superior specific capacity of 1002.5 mA h g^−1^ after 160 cycles at 0.2 C than the theoretical particular capacity of zinc oxide (978 mA h g^−1^). Hou et al. reported hydrothermally treated porous hard carbon materials.^[^ ^116^ ^]^ The porous structure was self‐templated by amorphous SiO_2_ nanoparticles incorporating raw RH and reached 679.9 mAh g^−1^ after 100 cycles at a current density of 0.2 C.

In any case, RH‐derived carbons have a high specific surface area, relusting in the formation of a huge solid electrolyte interphase (SEI) film, hence high properties have not been obtained. In the future, it may be possible to use iron‐catalytic graphitization to create graphitic carbon and SiO_2_ composites with low specific surface area.

#### 5.3.2 Supercapacitor

**Figure 7:**
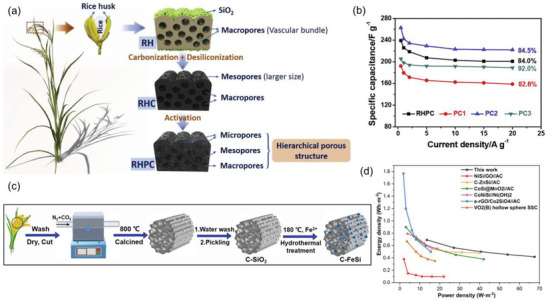
a and b) Graphical scheme of synthesis of hierarchical carbon from RHs and electrochemical performance as supercapacitors, respectively. The images were adapted from ref. [122] with permission from Elsevier, Copyright 2020. c and d) Graphical scheme of synthesizingiron‐doped hierarchical carbon from RHs and the electrochemical performance as a supercapacitor, respectively. The images were adapted from ref. [128] with permission from Elsevier, Copyright 2022.

The development of next‐generation sustainable supercapacitors urgently requires the search for renewable, cost‐effective, and environmentally friendly electrode materials capable of high adsorption, fast ion and electron transport, and surface chemistry tuning. In recent years, carbon electrode materials derived from biomass have attracted considerable attention as the electrodes of supercapacitors because they are widely available, renewable, and inexpensive.^[^ ^117^, ^118^ ^]^ More importantly, using biomass‐specific uniform and precise biological structures as templates allows the fabrication of electrode materials with controlled and well‐defined shapes. Biomass‐derived carbon materials are suitable for electrochemical reaction processes, such as ion transfer and diffusion, because of their specific naturally ordered hierarchical structure and rich surface properties.^[^ ^119^, ^120^ ^]^ Kumagai et al. synthesized micro‐ and mesoporous activated carbons derived from RHs and beet sugars. The specific capacitance, evaluated by means of cyclic voltammetry at a scan rate of 5 mV s^−1^, was 106 F g^−1^ for the aqueous electrolyte and 114 F g^−1^ for the organic electrolyte.^[^ ^121^ ^]^ Chen et al. synthesized RH‐based hierarchical porous carbon for high‐performance supercapacitors.^[^ ^122^ ^]^ The hierarchical porous carbon reaches 58.8 F g^−1^ at 0.5 A g^−1^ because of its high specific surface area and good pore structure (**Figure** **7**a,b). The hydrothermal process improved the surface area and pore structure, resulting in superior capacitance. In addition, the solubility of metal ions and their sorption onto porous carbon are improved under high temperatures and pressures. Owing to its excellent capacitance, several researchers have employed the hydrothermal process to synthesize capacitive metal oxides on RH‐derived carbon.^[^ ^123^, ^124^, ^125^ ^]^ Shen et al. developed nanostructured *α*‐MnO_2_ on RH carbon using a facile hydrothermal method.^[^ ^126^ ^]^ The branched structure of *α*‐MnO_2_ provides a high surface area, similar to *Actiniaria*. A high capacity of 977.4 C g^−1^ and good retention of 81.3% after 3000 cycles were demonstrated by MnO_2_/carbon. Xiao et al. reported Co_3_O_4_ nanocrystals loaded onto an RH‐derived carbon skeleton for supercapacitors.^[^ ^127^ ^]^ The RH carbon was synthesized and activated via calcination with KOH, and SiO_2_ was dissolved from RH into KOH during the activation process. The Co_3_O_4_ and carbon composite showed 671 F g^−1^ at 0.5 A g^−1^ and 94.01% retention after 5000 cycles. Wang et al. synthesized cauliflower‐like Fe_2_SiO_4_ on hierarchical‐pore RHs using a hydrothermal process,^[^ ^128^ ^]^ and they employed a hydrothermal process to form Fe_2_SiO_4_ from the added metal ions and SiO_2_ derived from RH (Figure 7c,d). Therefore, although pre‐calcination at 800 °C with KHCO_3_ is required to prepare porous carbon, the removal process of SiO_2_ is not required. The composites demonstrated a good capacitance of 534 F g^−1^ at 0.5 A g^−1^.

#### 5.3.3 Electrocatalysts

**Figure 8:**
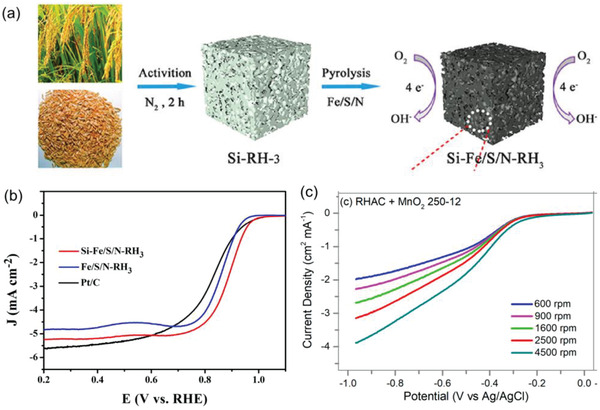
a and b) Graphical scheme of synthesis of heteroatom‐doped RH‐derived carbon and electrochemical performance as an electrocatalyst for the oxygen reduction reaction, respectively. The images were adapted and modified from ref. [142] with permission from Elsevier Copyright 2022. c) Electrochemical performance of RH‐derived carbon with metal oxides as electrocatalysts for the oxygen reduction reaction (ORR). The images were adapted from ref. [143] with permission from MDPI, Copyright 2020.

In fuel and metal‐air cells, the cathodic oxygen reduction reaction (ORR) is an essential factor determining energy conversion efficiency. To date, platinum‐based catalysts have been positioned as benchmarks for high‐performance electrode catalysts. However, platinum catalysts are expensive and less durable, and their rarity limits their large‐scale commercial use. For this reason, the design and manufacture of inexpensive and high‐performance nonprecious metal catalysts have been researched extensively. The most promising platinum‐free catalysts are transition metal/heteroatom‐doped carbon materials. Single, dual, and multiple doping with heteroatoms such as N, S, B, P, and metal ions leads to charge delocalization and asymmetric redistribution of the electron spin, which is experimentally and theoretically advantageous for the characteristics of ORR catalysts.^[^ ^129^, ^130^, ^131^, ^132^, ^133^, ^134^ ^]^ Biomass‐derived ORR electrodes have been used with HTC,^[^ ^120^, ^135^, ^136^, ^137^, ^138^, ^139^, ^140^ ^]^ where HTC facilitated heteroatom doping and low‐energy synthesis. In addition, composite materials, such as Fe and Co exhibited high activity in transition metals. Recently, heteroatom Si‐doped carbon catalysts were found to display higher catalytic activity than commercial Pt/C under alkaline conditions and improve the graphitization degree of the catalyst.^[^ ^141^ ^]^ Wang et al. reported the simple, easy, and scalable preparation of ORR catalysts based on self‐doped silicon and external confocal Fe, S, and N obtained from sustainable waste RH without the HTC process.^[^ ^142^ ^]^ The electrocatalysts demonstrated good ORR catalytic activity, particularly in Fe‐Nx active species, because the doped silicon in RH promoted the formation of pyridine N and graphite N formation. As a result, high power density and excellent durability were observed in Zn–air primary batteries when heteroatom‐doped carbon catalysts were used as air cathodes (**Figure** **8**a,b). Panganoron et al. employed HTC for several biomass materials, including RHs.^[^ ^143^ ^]^ The RHs were HTC‐treated with metal oxide *α*‐MnO_2_ at 250 °C for 12 h and demonstrated relatively high catalytic activity (Figure 8c). Although electrocatalysts that excluded additional annealing after HTC alone could not reach high activity levels because of their low conductivity, optimized HTC, such as high‐temperature HTC and mixing catalysts, is expected to increase the catalytic activity of RH carbon.

## 6 Conclusion and Future Perspectives

A vast amount of waste is generated as an agricultural by‐product in food production. Agricultural waste can be directly recycled into fertilizers and energy resources and through appropriate treatment, can become an effective value‐added resource. In addition, agricultural wastes can be transformed into unexpected value‐added materials for humans because of their complex and sophisticated structures and elemental compositions acquired from scientific research and technological development.

This review summarizes the separation and HTC of RHs for the development of high‐value‐added materials. Environmental applications of the obtained RHs, such as heavy metal adsorbents and energy devices, are also described. Various dry and wet separation methods have been considered for separating carbon and silica from RHs; however, many studies have concluded that treatment processes are expensive and complicated and have a high environmental impact. To effectively utilize biomass such as RHs, carbon and inorganic materials should be separated and recovered using a simple, low‐cost, and low environmental impact process. The HTC process can substantially reduce the cost of separation, resulting in value‐added RH. A careful redesign is required considering the processing load and benefits to be gained from applying HTC on RHs.^[^ ^144^ ^]^ As knowledge and technologies for separating and applying RHs accumulate, effective utilization of all parts of the rice plant should be promoted in the future. The HTC process contributes to the formation of a nanostructure with a high surface area; however, etching and heating processes are still needed to remove the amorphous SiO_2_ and transform hydrochars into conductive carbon. The sustainable process, combined with separation and carbonization, solves cumbersome processes.

## Conflict of Interest

The authors declare no conflict of interest.
